# Opportunities and challenges in utilizing community assets to extend chronic care management in podoconiosis endemic areas: Evidence from Northwestern Ethiopia

**DOI:** 10.1371/journal.pone.0309770

**Published:** 2024-10-22

**Authors:** Kibur Engdawork, Gail Davey, Getnet Tadele, Papreen Nahar, Shahaduz Zaman

**Affiliations:** 1 College of Social Sciences, Addis Ababa University, Addis Ababa, Ethiopia; 2 Centre for Global Health Research, Brighton and Sussex Medical School, United Kingdom; 3 School of Public Health, Addis Ababa University, Addis Ababa, Ethiopia; Ajou University School of Medicine and Graduate School of Medicine, REPUBLIC OF KOREA

## Abstract

Community-based chronic care intervention is important in the control and prevention of lifelong conditions such as podoconiosis and similar ‘intensive disease management’ neglected tropical diseases (NTDs). Despite a call for such interventions, few efforts have been made to identify opportunities and challenges related to their implementation. Employing a qualitative approach, this study explored the possibility of engaging community actors, physical places and networks to promote chronic care management in rural Ethiopia. We gathered data from affected individuals, community members, local leaders, health professionals and community health workers between April and May 2022. The study revealed that affected individuals, family members, religious leaders, traditional leaders, and health professionals have the potential to extend chronic care management in rural areas. Houses, churches, schools, and healthcare facilities are suitable venues at which to perform interventions. Strong community solidarity and political will are vital to chronic care interventions, while lack of understanding about chronic conditions, financial constraints, and limited resources at healthcare facilities could pose challenges. Using community assets has great promise for expanding chronic care management with minimal costs and efforts in under resourced areas. Achieving this will require a successful plan to coordinate the collaboration among the agents and settings.

## Introduction

Chronic conditions are defined by WHO as diseases of long duration and generally slow progression, not passed from one person to another and occurring a result of a combination of genetic, psychological, environment and behaviour factors [1]. The conditions require lifelong disease management to prevent physical pain, limit disability and support rehabilitation efforts [2]. However, despite their rising prevalence, affected individuals have poor self-care understanding and skill [3]. Adoption of long-term self-care may be difficult due to lack of accurate understanding, lack of self-discipline in adherence to self-care management, absence of a supportive environment, financial problems, or poor local health care systems [4, 5].

Podoconiosis is a chronic condition that needs lifelong care. It is a Neglected Tropical Disease (NTD) and a significant contributor to lymphoedema cases in tropical countries [6]. Although podoconiosis is not recognized as a killer disease, it causes severe swelling of the lower legs among barefoot walkers [7]. Podoconiosis develops when naturally susceptible individuals have chronic contact with red clay soil [7, 8]. The risk of developing podoconiosis can be averted by regular shoe wearing, consistent foot hygiene and covering floors [7, 9].

Ethiopia is the leading country globally in terms of burden of podoconiosis, with an estimated 1.5 million patients [10] and 35 million people at risk [11]. Podoconiosis accounts for the loss of 172,073 disability-adjusted years (DALYs) to Ethiopians annually [10]. Affected individuals have a lower quality of life compared with unaffected individuals. Podoconiosis interacts with determinants such as lack of formal education, living in urban areas, being single and having comorbidities to decrease affected individuals’ quality of life [12]. The physical injury and disability caused by podoconiosis result in depression among patients [13] and are associated with intense social stigma [14]. Research and advocacy workshops have yielded positive outcomes that include the decision of the Ethiopian government to incorporate podoconiosis intervention into national health programs and services. However, the country still has a long way to go to control podoconiosis [15].

The chronic care model developed by Wagner and the MacColl Institute for Healthcare Innovation aims to transform the daily care of patients with chronic conditions from acute to proactive, population-based and long-term disease management [16]. The model identifies community structures as essential elements along with health systems, self-management support, delivery system design, decision support and clinical information systems [16]. In LMICs, where healthcare worker ratios are low, community-based healthcare approaches may be the best way to assure adoption of self-care management [17].

WHO underlines the need for community participation to achieve the Sustainable Development Goals (SDGs) and calls for efforts to design strategies for community engagement [18]. Community engagement is understood as a process of developing and strengthening relationships that enable stakeholders to work together to address health-related issues [19]. This includes identifying change agents, individuals such as health professionals, teachers, local leaders, patients and community organizations, who are interested in promoting health and influencing health behaviour [18]. However, identifying these groups and organizations can not alone bring better health outcomes as it shall be supported by efforts to coordinate the activities of the groups and organizations. The community action theory posits that a successful coordination of activities among diverse organizations and groups within the community who agree to work together to achieve a common goal would help bring sustainable changes and provide a framework to structure and develop coalitions [20]. Social networks can be a key factor in determining how healthy a community is as they can create social supports that can help individuals to effectively cope with the impact of ill health [21]. Such efforts shall consider the diverse factors that influence the formation, implementation and maintenance of community collaborations [20].

Chronic conditions such as osteoarthritis, podoconiosis, leprosy and lymphatic filariasis (LF) have long-term consequences including mobility problems, disability, mental health and stigma. The conditions have high disease prevalence and burden but not necessarily have a high impact on mortality. As a result, they have been largely neglected by policy makers and intervention implementers [6, 22–25]. Chronic conditions are likely to require sustainable community-based interventions to enhance health systems and address socio-economic conditions and cultural contexts that entrap affected individuals [26, 27]. The effect of integrated disease management and community-based intervention in controlling skin NTDs in LMICs has been positive. Prior studies in India [28] and Ethiopia [29] showed that community-based interventions are effective in reducing acute attacks and stalling progress of lymphoedema. Recent research assessed an integrated lymphatic filariasis, podoconiosis and leprosy care package in Ethiopia and found it to be effective in improving lymphoedema, reducing morbidity and enhancing social support [13, 30]. Engaging the community in holistic care interventions for health problems caused by these conditions is promising in terms of improved health outcomes [31]. Utilizing community assets has been reported to improve the lives of people living with HIV in Ethiopia [32]. However, a prior study in Southern Ethiopia examined a podoconiosis intervention using the chronic care model and indicated its limitations in operationalizing measuring outcomes in quantifiable terms [33].

The goal of community-based intervention goes beyond enhancing people’s understanding and institutionalization of treatment activities in healthcare settings. Community-based interventions are designed to bring about changes in community health norms to prevent occurrence of conditions and reduce their impacts [34]. Despite increasing interest in community-based engagement, few empirical studies exist about what community assets can be utilized to introduce and sustain chronic care practices in endemic areas of podoconiosis and similar NTDs. We conducted a qualitative study in two podoconiosis-endemic districts of Northwestern Ethiopia to identify community assets such as agents, physical structure or places and informal networks in rural communities that might facilitate adoption of chronic care models in rural communities. Identifying the opportunities and challenges of utilizing community assets helps to understand how best chronic care intervention can be extended in low resource settings.

## Methods

### Setting and study population

We conducted a qualitative study in two rural communities in Amhara region, the second largest region in Ethiopia with high prevalence of podoconiosis. The prevalence of podoconiosis in the region is about 4%, with 64 of 164 districts identified as podoconiosis-endemic [11]. We purposefully selected two districts with 10% prevalence: Yilmana Densa in West Gojjam and Dera in South Gondar Zones of Amhara egion. Two non-governmental organizations (International Orthodox Christian Charities (IOCC) and National Podoconiosis Action Network (NaPAN)) conducted interventions to improve the lives of affected individuals in these communities. We evaluated the effectiveness of the intervention in improving the lives of individuals affected by podoconiosis. Data, informing this study, was collected during this evaluation study. Four *Kebeles* (administrative units) with around 6,400 households were purposefully selected for their high prevalence. There were 314 affected individuals living in these *Kebeles*.

### Study design

We conducted a qualitative study employing in-depth interviews, focus group discussions, observation and key informant interviews between April and May 2022. The study involved community members who were in the age category of 18 and 64. The qualitative data were collected by the lead author who has a PhD in sociology and a research assistant with a master’s degree in social psychology. Both researchers have extensive experience in conducting qualitative studies in podoconiosis and did not have any working relationship with intervention implementing NGOs.

We targeted affected individuals whose age was between 18 and 64, who were permanently living in the study area and who were willing to take part in the study. To capture maximum variation, we involved both genders and community members with various levels of education and economic status. We selected 32 affected individuals, fourteen of whom were females. Only four of the affected individuals had formal education and 28 of them were engaged in agricultural activities. We developed interview guides prior to the fieldwork to assess individuals’ access to healthcare services, interaction with other individuals, access to community resources and involvement in traditional institutions (see S1 File). We visited affected individuals at least twice at their homes. We began interviewing participants after initial visits, taking part in christening, festivities, and other social gatherings in respondents’ neighbourhoods to build a rapport. Interviews were conducted privately in the compound of affected individuals.

We also conducted Four Focus Group Discussions (FGDs) to elicit views about community assets from unaffected individuals in the two districts. Six and eight men participated in the FGDs conducted in Yilmana Densa and Dera districts, respectively. Eight women took part in two FGDs in the districts. We conducted group discussions at healthcare facilities on Sundays. These discussions were recorded and took about two hours.

We also conducted observations at households, healthcare facilities, churches and schools in the study settings. The observations enabled identification of facilities and resources that might be utilized for chronic care management interventions. We took notes during each observation, which were expanded into field notes each day after the completion of the observation.

We also interviewed 20 Key informants including staff of the implementing NGOs, health professionals and community health workers (widely known as health extension workers (HEWs) in Ethiopia) and local administrators. Interview guidelines were prepared to cover a range of themes about community resources, structures and change agents. Key informant interviews were conducted in the offices of respondents and recorded.

### Data analysis

Interviews and discussions were transcribed and translated into English. Observation field notes were translated into English. Transcripts were imported into NVivo version 12 software and were coded line by line into nodes. Data were structured into small pieces in line with the study objectives: what community assets exist in the communities and what are the opportunities and challenges associated with utilizing these community assets to extend chronic care intervention. The original nodes were regrouped and redefined under major themes to reflect information that emerged repeatedly and was deemed to be significant by the lead author (see S1 File). The lead author prepared a codebook and shared it with other authors. Research team members resolved overlapping themes and finally agreed on the codes and themes. Upon completion of each interview and discussion, we summarized the main points and read them back to our interviewees for confirmation. All FGD participants approved the summary. Most key informants and in-depth interview participants confirmed the data while some of the respondents provided additional information. To substantiate our arguments, we provide verbatim quotations as evidence.

### Ethical consideration

The study received ethical approval from the Research Governance and Ethics Committee (RGEC) of Brighton and Sussex Medical School (BSMS) (Reference: ER/BSMS9E3G/8) and the Ethiopian Society of Sociologists, Social Workers and Anthropologists (ESSSWA) (Reference: ESSSWA 019/21).

All participants agreed to take part in the study. Written informed consent was obtained from the participants. We read out consent forms for each participant and they confirmed their voluntary participation in the study by signing on consent forms. Preliterate participants confirmed voluntary participation by thumbprints. No safety incidents occurred to participants or researchers. We offered 200 ETB ($5) to participants to compensate their time.

Confidentiality in participating in the study was assured. Participant names and personal data were not included in the study. Each participant was assigned a study ID number, and their identity was not linked to the numbers. Interview transcripts are stored in the project SharePoint folders and have only been accessible to the research team.

## Results

### Community assets, and opportunities and challenges in utilizing them for community based interventions

Study participants indicated that affected individuals, family members, community leaders, traditional healers and health professionals are important influencers of health behaviour. The research team also observed that houses, traditional associations, churches, schools, and healthcare facilities are potentially suitable places and platforms to provide chronic care management interventions in rural areas.

### Agents

In collaboration with community health workers, we identified 314 individuals affected by podoconiosis in the study settings. Most of them had limited access to medical treatments and were at risk of developing complications. Family members of patients are among the important influencers of health, with affected individuals reporting that they relied on family members for emotional and physical support. An affected man stated, “My brothers are sympathetic to my condition. They helped me with agricultural and other activities” (IDI, affected man, age 50). Another affected man said, “My children and my wife take care of me. They wash my feet and suffer with me whenever I get sick. My children would go to the town and buy me pain killers. My wife washes my leg. I become grumpy whenever I have an acute attack, but they tolerate my behaviour” (IDI, affected male, age 56).

Utilizing family members to expand chronic care management in households may be challenged by existing gender roles. Although family members help patients, affected females do not get the same care and treatment as affected males. Affected females are still expected to perform their domestic tasks even when they are sick. An affected woman said, “Whenever I have an acute attack, my husband and children wait till I feel better instead of helping me with my household chores. They would rather go hungry for a day than cook for themselves” (Affected woman, age 45). Another affected woman added, “My husband and I are affected by podoconiosis. Whenever he has acute attacks, he sends our children to buy him painkillers. But, whenever I get sick, I sleep it off. My husband and children expect that I would eventually get well without any medication” (IDI, affected woman, age 38).

Key informants reported that community leaders, traditional healers and religious leaders played an important role in prior interventions in the communities. Health intervention implementers had collaborated with these agents to convince community members to utilize maternal health services. A health extension worker said, “We engage religious and community leaders to extend health information to community members. They are regarded as influential figures. Members respect and implement activities if they are approached via these individuals” (KII, HEWs, Dera district).

Traditional healers are also among the major agents that influence community members’ health behaviour. Some affected individuals reported visiting traditional healers to seek podoconiosis treatment. We found two traditional healers that provided treatment services at their houses. We observed that the traditional healers are perceived by community members to have the skills to treat many conditions. An affected individual said, “My parents took me to a traditional healer for treatment when I developed podoconiosis. He applied herbal medicine and the pimples on my foot completely disappeared” (IDI, 25 years old affected male).

Both traditional healers reported that they could not cure podoconiosis, but they treated patients to reduce the swelling and dry their wounds. Similar to lymphoedema management guidelines, the traditional healers advise affected individuals to wash their feet on a regular basis. A traditional healer said, “To reduce the swelling, I use herbal medicine to wash patients’ feet. I mix the herb with cows’ urine and soak it for a while and apply it on the swollen feet. However, if that does not work, I will move on to the next level of treatment by piercing their feet at various spots with a blade. Then, I will advise them to rinse and soak their feet in the hot spring” (KII, traditional healer, Dera district).

FGD participants and affected individuals also indicated that local health professionals and HEWs are important influencers of the health behaviour of rural residents. The health professionals and HEWs have frequent contact with affected individuals and the wider community. In particular, HEWs have constant contact with the community as they visit residents on a regular basis. We observed that HEWs provide health and counselling services to residents at the household level. NGOs working on podoconiosis interventions also utilized HEWs to recruit affected individuals for health interventions. A staff of an NGO stated that “We provided training for local health professionals at healthcare facility levels. We oriented these health professionals to train HEWs to enable them to provide health education. As HEWs have close contact with community members, using them as extenders of health information is advantageous” (KII, staff of IOCC).

### Physical structures and places

We observed that physical structures such as the homes of affected individuals, health posts, schools, health care facilities and churches could be suitable places to conduct chronic care interventions. Although there was variation in size and structure, we noted that everyone in the community owned a house. We observed that there were enough spaces to conduct health education and practice self-care management in affected individuals’ houses. The HEWs consult community members inside residents’ compounds. Individuals perform household chores and social activities at their homes. We further noticed that community members host social events at their houses and invitees discuss social and health issues during these events.

Several churches and two primary schools were found in the study areas. We noticed that the churches were accessible and had resources such as halls and verandas used to conduct health education interventions. We saw large groups of people gather at church every weekend. A prior health intervention targeted churches to recruit participants for health intervention on podoconiosis. A key informant noted, “Trained staff and HEWs provided health education to the community about podoconiosis at churches. That helped reach a large number of people in the community” (KII, NTD officer, Dera district). Utilizing religious organizations for health education intervention may face some challenges. Religious explanations of diseases may sometimes contradict health promotion messages. One affected individual said, “We can’t prevent anything if it comes from God. Anything can happen anytime by the will of God” (IDI, affected male, age 63).

There are also two primary schools in the study areas. FGD participants revealed that most children in the communities attend these schools. The schools have sufficient rooms and open spaces to conduct health education activities. NGOs operating in the communities to control podoconiosis underlined the importance of utilizing schools to conduct health education interventions. A key informant said, “Schools are ideal places to provide health interventions. We distributed medicine for diseases like trachoma and conducted health education intervention at nearby schools. You can easily get students and most of them are willing to take part in health interventions” (KII, HEW, Dera District). On the other hand, key informants emphasized that providing chronic care interventions at schools may not be sustainable unless integrated into the school system through meaningful engagement with teachers and students. A health extension worker noted, “We sometimes go to schools and conduct health campaigns. But those campaigns usually last for a few days; and lessons can be forgotten easily. We should engage teachers and school clubs to reach the wider student population” (KII, Health professional, Dera District). Health professionals shared their experience of how lack of collaboration with teachers resulted in unsuccessful attempts to administer COVID-19 vaccination in the communities. A health extension worker said, “The community was very hesitant to get COVID-19 vaccination as teachers warned members and students that the vaccines were not safe. The teachers browsed social media and spread false rumours about the vaccine. That made our campaign very difficult as many people believed the teachers” (KII, HEW, Yilmanana Densa District).

Key informants reported that local healthcare facilities were suitable to conduct health interventions. Each administrative unit in the two districts has one health post. The rooms are about 2m wide and 3m long and are furnished with three chairs and a table. Health extension workers spend some time at health posts providing health education, immunization, and curative services. Two health extension workers are assigned at each health post and serve as a primary point of entry to the health care system. The HEWs reported that health posts serve about 1,600 households in the study settings. The health posts are more accessible in terms of distance compared to other health care facilities. There were two healthcare facilities in the communities, constructed to better standards than the local housing. The premises were sufficiently large and contained neonatal intensive care, delivery, TB treatment, examination, emergency, laboratory, drug dispensary and management rooms and waiting areas. Key informants stated that the health care facilities serve about 40,000 residents of the surrounding *kebeles*, and operated with one physician, five health officers, five midwives, two laboratory technicians, eight clinical nurses, three pharmacists, and three administrative staff.

Community members paid regular visits to the healthcare facilities to get treatments. A FGD discussant reported, “Most of us now go to health centres when we are sick and for delivery. We also go to health posts to get family planning counselling” (FGD, Women, Yilmana Densa District). Most of the residents are enrolled in a community-based health insurance scheme. The participants believed that the scheme has improved access to health services as it enabled them to visit healthcare facilities on a regular basis without thinking of catastrophic expenses.

Prior health interventions have utilized these health care facilities and the health professionals to provide different health interventions. Key informants reported that the healthcare facilities served as venues of a podoconiosis intervention implemented by NGOs in 2021. The health professionals in the healthcare facilities took capacity development training and implemented interventions against podoconiosis. Many affected individuals came to the healthcare facilities to take part in the intervention. A health professional reported, “When there was an intervention on podoconiosis, many affected individuals, including those with swollen legs came to the stations to receive treatment” (KII, health professional, Yilmana Densa District). During the intervention, the health staff demonstrated to affected individuals how to wash and treat their feet. An affected person noted, “The health staff soaked our feet in water in a plastic bowl and showed us how to wash our feet” (IDI, a 25-year-old affected male). Along with introducing self-care and treatment procedures to patients, the foot washing demonstration sessions created an opportunity to put across a message to patients and to the community that the disease is not contagious. This was reflected in the following excerpt from an FGD participant “I have seen health professionals washing affected individuals’ feet, touching them with their hands. It gave me the lesson that the disease is not transmittable with skin contact” (FGD participant, male group, Dera district).

Utilizing local healthcare facilities and posts to provide chronic care services needs a strategy to sustainably integrate health service into the structure. Although about 10% of the population was affected by podoconiosis, FGD participants and some health professionals did not consider podoconiosis to be a prevalent health problem as FGD participants ranked TB, trachoma, malaria as the most prevalent health conditions that need attention. The healthcare facilities do not provide long term treatment or health literacy services to improve the chronic care skills and self-efficacy of affected individuals. A health professional noted, “We usually administer medicine for acute diseases. Even when podoconiosis patients visit us when they have acute attacks, we give them antibiotics and advise them to keep their personal hygiene. We don’t follow their progress and we only try to treat their acute attacks if they come to our healthcare facility” (KII, health officers, Dera district).

### Affected individuals’ understanding of podoconiosis and willingness to take part in health interventions

Community members indicated that affected individuals have a better understanding about podoconiosis and could disseminate health information in rural settings. An FGD discussant said, “Affected individuals tell us about the disease. They went to healthcare facilities and received health education. As they know the disease very well, they tell us how we can protect ourselves” (FGD, women, Dera district). Affected individuals reported that they tended to share health information with others. An affected woman said, “I am already affected by podoconiosis. I don’t want my children to suffer from the disease. So, I always tell them to wash their feet and wear shoes” (IDI, affected female, age 39).

Furthermore, affected individuals were eager to take part in health interventions and practice self-care management. Most affected individuals were willing to take part in the earlier NGO health intervention. These individuals appeared for self-care management training and adhered to self-care practices; some were able to improve their physical health. An affected male stated, “We have taken a lesson from health professionals on how to care for ourselves. We were informed by health professionals that we should continue applying the treatment procedures at home. And I have been practicing self-care (IDI, affected male, age 60).

The attempt to utilize affected individuals could face challenges. Some affected individuals, who had been involved in prior health interventions, wished to see immediate and complete changes in their physical condition and gave up on selfcare management when they realized that changes cannot readily happen. One affected male said, “Frankly speaking, the benefit of taking part in health intervention is not so promising. There is only a slight change in the swelling. I gave up on the treatment activities and I don’t usually practice the elevation exercise” (IDI, affected male, age 43).

Many affected individuals have limited physical movement affecting their productive capacity and by extension, their attempts to improve their living conditions. The physical impact of podoconiosis and poor economic status of affected individuals could hamper the utilization of affected individuals as purveyors of chronic care management. An affected person said, “The disease is painful and sometimes it keeps me at home. During the farming season it leaves me behind from preparing the farmland for the next harvest. Missing out that period affects my economic productivity. I also found it difficult to take part in social gatherings and other social activities” (IDI, male, age 50).

### Presence of established community social support structures and social networking

We observed strong social bonds among community members in the two study areas. Members of the two communities have a habit of sharing and celebrating events together. A number of social and work-related actions are performed together, and food and materials are shared with the less fortunate. An FGD participant said, “Sharing is a norm in our culture, it is a longstanding norm passed on from generation to generation” (FGD, Women Yilmana Densa District). This has created a feeling of connectedness between individuals in the communities. Most affected individuals believed that their neighbours and relatives care for them and would get their support in times of need.

Traditional institutions exist that are suitable to conduct health education interventions in the communities. Most study participants were members of traditional institutions such as *idir* and *mahiber*. The former is a self-help voluntary association that serves as economic and social insurance at times of death and crises while the latter is a family and religious-based association wherein members meet once a month to commemorate saints. Beside their manifest functions, the traditional associations serve as platforms to discuss social issues and strengthen the solidarity of members. FGD participants believed that membership in such associations was helpful when coping with difficult times such as the death of a family member. Currently, *idirs* only provide help to members during crises, but not during sickness or other needs. A participant in a FGD said, *“Idir* is a crucial social institution which is useful to strengthen our social bonds. It serves as social insurance for the poor. In time of death (mourning), *idir* assists the deceased family equally regardless of their wealth status” (FGD, Women Yilmana Densa District). Another FGD participant added, “We commemorate saints monthly under our *mahibers*. On top of performing religious rituals, we talk about our personal problems when we meet” (FGD, women, Dera District).

Most affected individuals were members of such associations. Having podoconiosis did not affect membership as long as affected individuals were capable of paying membership fees. An affected person said, “I am a member of the association that honours Saint Gabriel. I am the only patient in our association. The association has around 40 members. I usually participate, except when I have acute attacks… It is helpful specially in time of harvest and farming or building a house. Whenever I need help from members, they are willing to assist me” (IDI, a 57-year-old affected male).

### The religiosity of the community

Almost all participants of the study were followers of Orthodox Christianity. Religion is highly revered and shapes residents’ beliefs. We noted that most people went to church on a regular basis to perform religious duties. FGD participants revealed that residents observe fasts and go to church every weekend. An affected community member said “I am a farmer. …I wake up early in the morning and go to church to praise the Lord…This is a very important thing for me. I do this every morning” (IDI, affected female, age 64).

Most participants also have a tendency to explain things including disease in supernatural terms and rely on religion to cope with their daily challenges. One example includes: “The Lord gives, and the Lord takes away. God gave me a beautiful foot at birth, and HE took it away. I have not taken remorse against God. I thank my God because HE also gave me the strength to cope up the challenges” (IDI, affected female, age 35 to 38 years old).

The religiosity of the community is well noted by implementers and attempts have been made to implement interventions through religious organizations to alter people’s health-related behaviour. Religious leaders are among the most respected people in the community. Most key informants held the opinion that utilizing religious leaders could yield a better outcome in podoconiosis prevention and control. A key informant noted, “A well-planned community campaign involving religious leaders might be crucial to achieve community-wide adherence to treatments and prevention practices” (KII, staff of an NGO).

The religiosity of the community could also contribute to lesser stigma towards affected individuals. It appears that the communities to some extent tolerated infirmities like podoconiosis. The communities did not seem to apply any sanctions to affected individuals. Affected individuals took part in several social and religious activities. The mere presence of negative reactions towards affected individuals seemed to relate to the community’s belief that the condition is not the result of personal choice. Community members reported that it is their religious duty to care for the sick and needy. FGD participants said that God had power over the disease and stigmatizing against patients could result in having similar or other diseases as punishment. An affected person said, “I have not encountered any problem with regards to my illness. This is just a disease that could have happened to anyone in the community” (IDI, affected female, age 64).

### Experience of community-based interventions

We further learned that prior efforts to engage community assets have brought desirable health benefits in the communities. For example, FGD participants reported that women in the community started giving birth at healthcare facilities following health education and follow-up by HEWs. A discussant in FGD said, “Many women used to give birth at home, and some died due to heavy bleeding during labour. This has been changed now as women give birth at health centres” (FGD, Women Yilmana Densa District).

The communities also successfully combated open defecation practices through community-based intervention. According to the key informants, defecating in open areas had been a long-standing practice and was so common that a mere health education program could not bring it to an end. Intervention implementers then came up with a community sensitization strategy. They recruited community representatives; the clergy, local leaders, influential community representatives and HEWs, to form a local committee. The committee then selected model household heads and provided them with training and support to construct a latrine in their compound. As part of the interventions, these individuals established a committee of 30 to 40 individuals. These individuals were invited to visit the newly built latrines and were asked to construct the same. The established groups then crafted their own rules to enforce the construction of latrines. The rules included fines for those who failed to build the latrines. The group members also were assigned with the task of naming and shaming those who practiced open defecation in the communities. Religious leaders also taught about the importance of using toilets and reprimanded those who failed to construct latrines and did not use toilets. The HEWs went door to door to teach people about the consequences of open defecation on children’s and adult’s health. This effort was very successful in ending open defecation, local health professionals remarked. A discussant in FGD reported, “Back in the days we used to defecate wherever we wanted. Now we even ridicule people who defecate in the fields. We do use latrines nowadays. (FGD, Men Yilman Densa).

### Political commitment to implement interventions against podoconiosis

In line with the Ethiopian government’s plan to fight podoconiosis, the regional and district level government units showed a strong will to collaborate with intervention implementers and to set up government structures to provide services for podoconiosis patients. A key informant said, “The NTD section has been established in the health sector structure at regional, zonal and district level to account for podoconiosis” (KII, NTD officer, Yilmana Densa District). The government units and employees also collaborated with health intervention implementers. A staff of intervention implementers noted, “Regional offices always collaborate with us. This has enabled sharing, knowledge, resources and experience, and minimized resource duplications” (KII, staff of intervention implementing organization).

Although the Ethiopian government has established NTD structures at district level, this commitment has not been backed by allocation of resources. As a result, prior attempts to mainstream podoconiosis services into local healthcare facilities faced major challenges. Our study noted that although a prior intervention by NGOs introduced lymphoedema services for podoconiosis patients, they could not be continued in the healthcare facilities. A health professional also said, “We once provided health resources and treatment for podoconiosis patients for three months. However, we could not continue the services as our healthcare service is not receiving support from the health bureau” (KII, health professional, Yilmana Densa District). An affected female further reported, “Although I went to a healthcare facility this year and asked the health staff to register me for support, they informed me that it was too late, and no service was provided at that time” (IDI, an affected female, age 38).

## Discussion

Our study indicated that affected individuals, family members, community associations, schools, religious institutions and healthcare centres and professionals hold powerful potential to expand chronic care management. Engaging these community assets in chronic care interventions requires smooth coordination of intervention activities. Community-based programs in Sub-Saharan Africa have not been consistent as they have lacked coordinated responses and face context-specific challenges [17]. These community assets are connected in social networks. The existing relationship helped individuals to cope with their social problems. Existing networks also helped to create a strong bond and trust among community members. The networks have been used to bring normative changes in the communities; thereby indicating the possibility of using community assets as a force for community improvement in chronic care management.

Utilizing community assets for chronic care management to improve prevention against podoconiosis and similar NTDs in Ethiopia and similar under-resourced settings is not well-charted territory. The approach not only needs to involve affected individuals but also the wider community and local contexts. Engaging community assets in health intervention may help tackle the social causes of ill health and strengthen community involvement in health intervention. If implementers work with community assets, it may be possible to enhance individuals’ knowledge and skill and improve the socio-economic status of affected individuals and at-risk population. This may help to change community norms that contribute to ill health. This could go beyond fighting specific prevalent diseases and create a resilient community to health and social problems.

Enhancing affected individuals’ disease management skills can improve their health. Chronic patients are experts in handling their health and life [35]. However, experiencing chronic conditions may not always improve understanding of treatment activities [36]. Thus, any interventions must enhance the skills of affected individuals in chronic care management. Trained affected individuals should be active contributors to their own care, whereas local health professionals should facilitate the care [36].

Affected individuals’ knowledge and abilities have the potential to transform chronic care management in rural communities. Our study revealed that affected individuals who had participated in a prior health intervention had better understanding of podoconiosis and having experienced the disease, were willing to take part in future chronic care interventions. This indicates the possibility of utilizing affected individuals as extenders of chronic care management lessons in under-resourced settings. An intervention in Southern Ethiopia successfully utilized patients to disseminate information about podoconiosis and detect patients in rural areas [33]. Studies that focused on HIV/AIDS in Africa also showed that employing people living with HIV to extend health information can be effective as audiences find messages by people with HIV to be trustworthy and convincing [37, 38].

Affected individuals’ experiences in health interventions could increase the commitment of individuals to join collective efforts of fighting health conditions as noted by Butterfoss and Kegler [20]. Utilizing patients in chronic care management interventions will still require several barriers to be overcome. Some affected individuals had little understanding about chronic care and felt that their needs were not met by prior medical interventions, resulting in them dropping out. Like an earlier study [39], we noted that affected individuals discontinued treatment activities after realizing that they did not immediately improve their physical health. Intervention implementors need to help affected individuals accept their chronic condition and enhance their self-efficacy to adopt chronic care. The provision of training may help patients realize that podoconiosis is a lifelong condition, but that they can manage their condition to improve their overall health. Affected individuals have other identities and skills; emphasising these aspects could improve their confidence and ability to make health related decisions. People’s economic condition was an important factor shaping their adherence to preventive actions, as noted previously [39]. Interventions must also provide resources for those with financial constraints to purchase self-management items. Unless intervention prioritizing building the economic capacity of affected individuals, other efforts may not result in meaningful improvements. Prior studies in Ethiopia have shown the importance of enhancing the livelihoods of target individuals and at-risk communities to sustain intervention efforts [38, 40]. Increasing access to credit services and resources for agricultural and small business activities would enhance the economic status of residents in such communities.

Engaging community health workers may help sustain chronic care management in rural areas. The Ethiopian Health Extension Program is suitable to accommodate lessons related to chronic care management through hygiene and environmental sanitation, disease prevention and control and health education package. Supported by a supervisory framework involving federal, regional, and woreda (district) health offices, HEWs provide the services at healthcare posts and other community venues [41]. Any attempt that aims to utilize HEWs shall consider mainstreaming activities and services into existing packages and strengthen collaboration between HEWs and other healthcare workers. HEWs demonstrated willingness to take capacity building training and treat affected individuals. A study showed that HEWs enhanced rural youth’s literacy about podoconiosis in Southern Ethiopia [42] indicating the possibility of engaging HEWs in enhancing chronic care intervention. Utilizing HEWs as extenders of chronic care management, however, should go beyond the traditional diffusion of health information, and help community members adopt chronic care skills. Implementers will also need to provide training on how to supervise affected individuals’ self-care. This training needs to be backed by the provision of devices such as tablets and software to record and share patient data. In addition, since HEWs are often overburdened with prevalent communicable diseases and reproductive health services, intervention implementers will need to motivate them with financial incentives and material support.

This study indicated the possibility of using traditional healers to help the community adopt chronic care management skills. This is in line with the recommendation of the WHO to integrate traditional healers into the healthcare system [43]. Our study showed that traditional healers recognize podoconiosis as a chronic condition. Due to their accurate understanding and valuable relationship with community members, they could be prominent propagators of chronic care management in rural areas. There is little evidence about collaboration between traditional healers and health intervention implementers to deliver a chronic care service for NTDs. Studies in Africa have shown that traditional healers are willing to detect cases and refer patients to local health care facilities [44, 45]. Rural residents may continue to visit traditional medicine centres. Thus, intervention implementers need to come up with a strategy to link the traditional healing system with existing healthcare structures to extend chronic care management skills.

Residential settings are increasingly used for public health interventions in developed countries [46]. Housing-based interventions could assist at-risk and affected individuals to cover their floor surfaces inside their traditional huts as recommended by WHO [9] and foster physical and social support for affected individuals. Rural houses are also used to host social gatherings and regular meetings of traditional associations. This social dimension of housing may be suitable to implement house-based health interventions.

Engaging community associations may support chronic care management interventions. Affected individuals actively participate in these community associations and have strong trust in fellow members. To date, there has been little evidence on whether and to what extent chronic health messages have been mainstreamed within community associations. Sustainable efforts to expand chronic care management could benefit from utilizing associations to bring normative changes. However, it should be noted that for a very long time, members of the communities have been pooling their resources to overcome economic and social difficulties. It is evident that these associations were not initially developed as health schemes. Intervention implementers should work with the associations to modify the institutions’ original purpose. A prior study showed that an intervention succeeded in changing traditional institutions from emergency groups into mutual aid organizations to mitigate the impact of HIV/AIDS in Ethiopia [47]. It is increasingly noted that these traditional institutions have started operating beyond their original objectives and offering psychosocial counselling and financial aid for people who live with HIV/AIDS [48].

Advocates of community-based interventions argue that health programs can be situated within healthcare institutions or other community structures [34]. Existing community venues such as churches might be suitable for chronic care interventions on podoconiosis in communities where members are regular churchgoers. Churches are suitable platforms to recruit and retain health intervention participants for an extended period of time [49]. However, implementers may disseminate health messages that may contradict religious explanations of disease. Sometimes supernatural explanations may impact the adoption of and adherence to preventive action if they lead to beliefs that diseases cannot be controlled by human actions. To avoid any potential clash, interventions will need to synchronize their messages with religious explanations. Following the COVID-19 outbreak, religious institutions collaborated with intervention implementors to convince people to adhere to preventive actions and get vaccinated [50]. Collaboration between religious, science and political leaders to fight diseases has a long history [51].

Health intervention implementers might provide information about chronic care management in schools. Our study showed that schools could be accessible places to conduct health promotion interventions as implementers can meet different categories of community members including students, teachers and parents on a regular basis. An earlier study in Ethiopia indicated that school-based strategies are low cost and brought changes in personal hygiene among students [52]. Providing training on chronic care for teachers and students could help reach the wider community.

We noted that utilizing existing healthcare facilities and health professionals would be suitable for provision of chronic care interventions. To promote chronic care, community agents and community structures need linkages with local health care structures. Indeed, health care facilities are important elements of the chronic care model [16]. Isolated information dissemination efforts which lack linkages with established health systems can be less effective [53]. Implementers should periodically conduct training workshops to enable healthcare facilities and health professionals to provide chronic care services. Additionally, interventions will need to back local healthcare facilities with materials and financial resources to sustain services. Developing and distributing materials on chronic care adapted to the needs of local healthcare facilities could facilitate the continuation of care. Conducting health education at healthcare facilities using a set of easy demonstrating techniques can enhance rural residents’ understanding of chronic care. Like a study in South Africa [54], our study noted that the healthcare facilities are more used to providing acute care services. As a result, conducting chronic care management intervention may require allocating new tasks to health professionals at healthcare facilities and health extension workers at health posts. Shifting tasks related to counselling services, training on self-management and follow up to health extension workers at health posts could lessen the burden on health professionals. Health officers, public health nurses, environmental/hygiene experts and health education experts operating at district levels could supervise HEW’s activities as part of their routine monitoring activities. Redistribution of tasks across members of healthcare facilities could be a viable and cost-effective solution to improving care for individuals with chronic conditions in primary care in LMICs [55].

This study showed that prior attempts to utilize community assets resulted in normative changes. The community’s experience with similar interventions is a critical factor that can affect the establishment of successful future collaborative relationships [20]. Identified community assets could be employed to adopt chronic care management for podoconiosis and similar conditions in rural areas. Chronic care frameworks recognize community assets in easing the burden of chronic care services [56]. Community-based interventions are reported to improve efforts of managing physical pain and enhance affected individuals’ ability to cope with the emotional and psychological challenges of chronic conditions [57]. Interventions should coordinate joint activities among these assets to reach affected individuals and populations at risk of podoconiosis. Periodic meetings could help share knowledge, skills, experience, and challenges of chronic care management among residents. The social capital and trust among community assets could affect the formation and function of community associations [58]. These efforts must be backed by policies to establish health issues as priorities in local government structures, provide funding, or encourage collaboration to make them for effective and sustainable.

This study has provided evidence on the possibility of utilizing community assets and networks in rural areas to implement chronic care management interventions to prevent and control podoconiosis. The study relied on prior experience and interventions in the community to draw lessons for future interventions. The study suggested utilizing preexisting networks in rural communities. The networks can further be used to address other community concerns and issues. We gathered data from a range of groups, resulting in rich and deep data. On the other hand, there were some limitations. The study focused on just two rural communities in Ethiopia. The findings may not be generalizable to other settings with different community structures. We have identified community assets as indicated by the study participants. The capacity of the community assets was not rigorously evaluated as we mainly relied on participants’ suggestions. Future studies should examine the cost-effectiveness of engaging community assets in chronic care intervention and their possible impact in controlling and preventing podoconiosis and similar NTDs.

## Supporting information

S1 File (DOCX)

## References

[1] Noncommunicable diseases [Internet]. 2023. Available from: https://www.who.int/news-room/fact-sheets/detail/noncommunicable-diseases.

[2] Beaglehole R, Epping-Jordan J, Patel V, Chopra M, Ebrahim S, Kidd M, et al. Improving the prevention and management of chronic disease in low-income and middle-income countries: a priority for primary health care. Lancet. 2008;372(9642):940–9. doi: 10.1016/S0140-6736(08)61404-X 18790317

[3] Chan SW. Chronic Disease Management, Self-Efficacy and Quality of Life. J Nurs Res. 2021;29(1):e129. doi: 10.1097/JNR.0000000000000422 33427791 PMC7808345

[4] Jerant AF, von Friederichs-Fitzwater MM, Moore M. Patients’ perceived barriers to active self-management of chronic conditions. Patient Educ Couns. 2005;57(3):300–7. doi: 10.1016/j.pec.2004.08.004 15893212

[5] Bayliss EA, Steiner JF, Fernald DH, Crane LA, Main DS. Descriptions of barriers to self-care by persons with comorbid chronic diseases. Ann Fam Med. 2003;1(1):15–21. doi: 10.1370/afm.4 15043175 PMC1466563

[6] Molyneux DH. Tropical lymphedemas—control and prevention. N Engl J Med. 2012;366(13):1169–71. doi: 10.1056/NEJMp1202011 22455411

[7] Price EW, Plant DA. The significance of particle size of soils as a risk factor in the etiology of podoconiosis. Trans R Soc Trop Med Hyg. 1990;84(6):885–6. doi: 10.1016/0035-9203(90)90115-u 2096529

[8] Davey G., Podoconiosis non-filarial elephantiasis, and lymphology. Lymphology. 2010;43(4):168–77. 21446572

[9] Deribe K, Cano J, Trueba ML, Newport MJ, Davey G. Global epidemiology of podoconiosis: A systematic review. PLoS Negl Trop Dis. 2018;12(3):e0006324. doi: 10.1371/journal.pntd.0006324 29494642 PMC5849362

[10] Deribe K, Negussu N, Newport MJ, Davey G, Turner HC. The health and economic burden of podoconiosis in Ethiopia. Trans R Soc Trop Med Hyg. 2020;114(4):284–92. doi: 10.1093/trstmh/traa003 32055853 PMC7139123

[11] Deribe K, Cano J, Newport MJ, Golding N, Pullan RL, Sime H, et al. Mapping and Modelling the Geographical Distribution and Environmental Limits of Podoconiosis in Ethiopia. PLoS Negl Trop Dis. 2015;9(7):e0003946. doi: 10.1371/journal.pntd.0003946 26222887 PMC4519246

[12] Mousley E, Deribe K, Tamiru A, Davey G. The impact of podoconiosis on quality of life in Northern Ethiopia. Health Qual Life Outcomes. 2013;11:122. doi: 10.1186/1477-7525-11-122 23866905 PMC3726315

[13] Ali O, Deribe K, Semrau M, Mengiste A, Kinfe M, Tesfaye A, et al. A cross-sectional study to evaluate depression and quality of life among patients with lymphoedema due to podoconiosis, lymphatic filariasis and leprosy. Trans R Soc Trop Med Hyg. 2020;114(12):983–94. doi: 10.1093/trstmh/traa130 33190154 PMC7738660

[14] Tora A, Franklin H, Deribe K, Reda AA, Davey G. Extent of podoconiosis-related stigma in Wolaita Zone, Southern Ethiopia: a cross-sectional study. Springerplus. 2014;3:647. doi: 10.1186/2193-1801-3-647 25485190 PMC4233027

[15] Mengitsu B, Shafi O, Kebede B, Kebede F, Worku DT, Herero M, et al. Ethiopia and its steps to mobilize resources to achieve 2020 elimination and control goals for neglected tropical diseases webs joined can tie a lion. Int Health. 2016;8 Suppl 1:i34–52. 26940308 10.1093/inthealth/ihw007

[16] Wagner EH, Austin BT, Davis C, Hindmarsh M, Schaefer J, Bonomi A. Improving chronic illness care: translating evidence into action. Health Aff (Millwood). 2001;20(6):64–78. doi: 10.1377/hlthaff.20.6.64 11816692

[17] Ochola EA, Karanja DMS, Elliott SJ. Local tips, global impact: community-driven measures as avenues of promoting inclusion in the control of neglected tropical diseases: a case study in Kenya. Infect Dis Poverty. 2022;11(1):88. doi: 10.1186/s40249-022-01011-w 35932055 PMC9356398

[18] Community engagement: a health promotion guide for universal health coverage in the hands of the people [Internet]. World Health Organization. 2020.

[19] WHO community engagement framework for quality, people-centred and resilient health services. [Internet]. WHO. 2017. Available from: https://iris.who.int/bitstream/handle/10665/259280/WHO-HIS-SDS-2017.15-eng.pdf?sequence=1.

[20] Butterfoss FD, Kegler MC. The community coalition action theory. Emerging theories in health promotion practice and research, 2nd ed. Hoboken, NJ, US: Jossey-Bass/Wiley; 2009. p. 237–76.

[21] Perkins JM, Subramanian SV, Christakis NA. Social networks and health: a systematic review of sociocentric network studies in low- and middle-income countries. Soc Sci Med. 2015;125:60–78. doi: 10.1016/j.socscimed.2014.08.019 25442969 PMC5690563

[22] Yahaya I, Wright T, Babatunde OO, Corp N, Helliwell T, Dikomitis L, et al. Prevalence of osteoarthritis in lower middle- and low-income countries: a systematic review and meta-analysis. Rheumatol Int. 2021;41(7):1221–31. doi: 10.1007/s00296-021-04838-y 33907879 PMC8164595

[23] Mitjà O, Marks M, Bertran L, Kollie K, Argaw D, Fahal AH, et al. Integrated Control and Management of Neglected Tropical Skin Diseases. PLoS Negl Trop Dis. 2017;11(1):e0005136. 10.1371/journal.pntd.0005136 PMC5245794 28103250

[24] Zeldenryk LM, Gray M, Speare R, Gordon S, Melrose W. The emerging story of disability associated with lymphatic filariasis: a critical review. PLoS Negl Trop Dis. 2011;5(12):e1366. doi: 10.1371/journal.pntd.0001366 22216361 PMC3246437

[25] van Brakel WH, Sihombing B, Djarir H, Beise K, Kusumawardhani L, Yulihane R, et al. Disability in people affected by leprosy: the role of impairment, activity, social participation, stigma and discrimination. Glob Health Action. 2012;5. doi: 10.3402/gha.v5i0.18394 22826694 PMC3402069

[26] Dean L, Theobald S, Nallo G, Bettee A, Kollie K, Tolhurst R. A syndemic born of war: Combining intersectionality and structural violence to explore the biosocial interactions of neglected tropical diseases, disability and mental distress in Liberia. PLOS Glob Public Health. 2022;2(6):e0000551. doi: 10.1371/journal.pgph.0000551 36962440 PMC10021464

[27] Mendenhall E, Kohrt BA, Norris SA, Ndetei D, Prabhakaran D. Non-communicable disease syndemics: poverty, depression, and diabetes among low-income populations. Lancet. 2017;389(10072):951–63. doi: 10.1016/S0140-6736(17)30402-6 28271846 PMC5491333

[28] Mues KE, Deming M, Kleinbaum DG, Budge PJ, Klein M, Leon JS, et al. Impact of a community-based lymphedema management program on episodes of Adenolymphangitis (ADLA) and lymphedema progression—Odisha State, India. PLoS Negl Trop Dis. 2014;8(9):e3140. doi: 10.1371/journal.pntd.0003140 25211334 PMC4161333

[29] Hounsome N, Kassahun MM, Ngari M, Berkley JA, Kivaya E, Njuguna P, et al. Cost-effectiveness and social outcomes of a community-based treatment for podoconiosis lymphoedema in the East Gojjam zone, Ethiopia. PLoS Negl Trop Dis. 2019;13(10):e0007780. doi: 10.1371/journal.pntd.0007780 31644556 PMC6808421

[30] Dellar R, Ali O, Kinfe M, Mengiste A, Davey G, Bremner S, et al. Effect of a Community-Based Holistic Care Package on Physical and Psychosocial Outcomes in People with Lower Limb Disorder Caused by Lymphatic Filariasis, Podoconiosis, and Leprosy in Ethiopia: Results from the EnDPoINT Pilot Cohort Study. Am J Trop Med Hyg. 2022;107(3):624–31. doi: 10.4269/ajtmh.21-1180 35895351 PMC9490655

[31] Tesfaye A, Semrau M, Ali O, Kinfe M, Tamiru M, Fekadu A, et al. Development of an integrated, holistic care package for people with lymphoedema for use at the level of the Primary Health Care Unit in Ethiopia. PLoS Negl Trop Dis. 2021;15(4):e0009332. doi: 10.1371/journal.pntd.0009332 33878110 PMC8086999

[32] Hussen SA, Tsegaye M, Argaw MG, Andes K, Gilliard D, del Rio C. Spirituality, social capital and service: factors promoting resilience among Expert Patients living with HIV in Ethiopia. Glob Public Health. 2014;9(3):286–98. doi: 10.1080/17441692.2014.880501 24520996 PMC4033693

[33] Davey G, Burridge E. Community-based control of a neglected tropical disease: the mossy foot treatment and prevention association. PLoS Negl Trop Dis. 2009;3(5):e424. doi: 10.1371/journal.pntd.0000424 19479039 PMC2682702

[34] Silver D. A brief history of community-based health interventions. In: Guttmacher S, Vana PK, Ruiz-Janecko Y, editors. Community-Based Health Interventions: Wiley; 2010.

[35] Meade MA, Cronin LA, editors. The Expert Patient and the Self-Management of Chronic Conditions and Disabilities2012.

[36] Badcott D. The expert patient: valid recognition or false hope? Med Health Care Philos. 2005;8(2):173–8. doi: 10.1007/s11019-005-2275-7 16215797

[37] Setel P. A Plague of Paradoxes: AIDS, Culture, and Demography in Northern Tanzania: University of Chicago Press; 1999.

[38] Tadele G, Ayalew A, Loevinsohn ME. (Re) building Livelihoods of Communities Confronting HIV and AIDS in Ethiopia. Eastern Africa Social Science Research Review. 2016;32:63–91.

[39] Tora A, Davey G, Tadele G. Factors related to discontinued clinic attendance by patients with podoconiosis in southern Ethiopia: a qualitative study. BMC Public Health. 2012;12:902. doi: 10.1186/1471-2458-12-902 23095311 PMC3503806

[40] Tora A, Davey G, Tadele G. A qualitative study on stigma and coping strategies of patients with podoconiosis in Wolaita zone, Southern Ethiopia. Int Health. 2011;3(3):176–81. doi: 10.1016/j.inhe.2011.06.006 24038367

[41] Wang H, Tesfaye R, Ramana GNV, Chekagn CT. Ethiopia Health Extension Program: An Institutionalized Community Approach for Universal Health Coverage: World Bank Publications; 2016.

[42] Engdawork K, McBride CM, Ayode D, Allen CG, Davey G, Tadele G. Rural youths’ understanding of gene x environmental contributors to heritable health conditions: The case of podoconiosis in Ethiopia. PLoS Negl Trop Dis. 2018;12(9):e0006763. doi: 10.1371/journal.pntd.0006763 30212466 PMC6155534

[43] WHO Traditional Medicine Strategy: 2014–2023 [Internet]. 2013.

[44] Audet CM, Clemens EM, Ngobeni S, Mkansi M, Sack DE, Wagner RG. Throwing the bones to diagnose HIV: Views of rural South African traditional healers on undertaking HIV counselling and testing. AIDS Care. 2021;33(10):1316–20. doi: 10.1080/09540121.2020.1808568 32799661 PMC7887123

[45] Kwedi Nolna S, Ntonè R, Fouda Mbarga N, Mbainda S, Mutangala W, Boua B, et al. Integration of Traditional Healers in Human African Trypanosomiasis Case Finding in Central Africa: A Quasi-Experimental Study. Trop Med Infect Dis. 2020;5(4). doi: 10.3390/tropicalmed5040172 33212918 PMC7709689

[46] Hernández D. Housing-Based Health Interventions: Harnessing the Social Utility of Housing to Promote Health. Am J Public Health. 2019;109(S2):S135–s6. doi: 10.2105/AJPH.2018.304914 30785797 PMC6383973

[47] Stuer F, Ogojo Okello F, Wube M, Steinitz LY. From Burial Societies to Mutual Aid Organizations: The Role of Idirs—Traditional Burial Societies in Ethiopia—In Ensuring Community-Level Care and Protection of Vulnerable Children. Journal of HIV/AIDS & Social Services. 2012;11:57–76.

[48] Pankhurst A, Tesfaye A, Gebre A, Tekola B, Demile H. Social responses to HIV/AIDS in Addis Ababa, Ethiopia with reference to commercial sex workers, people living with HIV/AIDS and community-based funeral associations in Addis Ababa. Organization for Social Science Research in Eastern and Southern Africa (Ed), The HIV/AIDS challenge in Africa: An impact and response assessment: The case of Ethiopia. 2008:149–332.

[49] Campbell MK, Hudson MA, Resnicow K, Blakeney N, Paxton A, Baskin M. Church-based health promotion interventions: evidence and lessons learned. Annu Rev Public Health. 2007;28:213–34. doi: 10.1146/annurev.publhealth.28.021406.144016 17155879

[50] Wijesinghe MSD, Ariyaratne VS, Gunawardana BMI, Rajapaksha R, Weerasinghe W, Gomez P, et al. Role of Religious Leaders in COVID-19 Prevention: A Community-Level Prevention Model in Sri Lanka. J Relig Health. 2022;61(1):687–702. doi: 10.1007/s10943-021-01463-8 34812996 PMC8609254

[51] Hong BA, Handal PJ. Science, Religion, Government, and SARS-CoV-2: A Time for Synergy. J Relig Health. 2020;59(5):2263–8. doi: 10.1007/s10943-020-01047-y 32488828 PMC7266421

[52] Appleby LJ, Tadesse G, Wuletawu Y, Dejene NG, Grimes JET, French MD, et al. Integrated delivery of school health interventions through the school platform: Investing for the future. PLoS Negl Trop Dis. 2019;13(1):e0006449. doi: 10.1371/journal.pntd.0006449 30703087 PMC6354954

[53] Jackson SF, Perkins F, Khandor E, Cordwell L, Hamann S, Buasai S. Integrated health promotion strategies: a contribution to tackling current and future health challenges. Health Promot Int. 2006;21 Suppl 1:75–83. doi: 10.1093/heapro/dal054 17307960

[54] Abrahams N, Gilson L, Levitt NS, Dave JA. Factors that influence patient empowerment in inpatient chronic care: early thoughts on a diabetes care intervention in South Africa. BMC Endocr Disord. 2019;19(1):133. 31806000 10.1186/s12902-019-0465-1 PMC6896266

[55] Seidman G, Atun R. Does task shifting yield cost savings and improve efficiency for health systems? A systematic review of evidence from low-income and middle-income countries. Hum Resour Health. 2017;15(1):29. doi: 10.1186/s12960-017-0200-9 28407810 PMC5390445

[56] Innovative Care for Chronic Conditions: Building blocks for action [Internet]. WHO. 2002.

[57] Coventry PA, Small N, Panagioti M, Adeyemi I, Bee P. Living with complexity; marshalling resources: a systematic review and qualitative meta-synthesis of lived experience of mental and physical multimorbidity. BMC Fam Pract. 2015;16:171. doi: 10.1186/s12875-015-0345-3 26597934 PMC4657350

[58] Wolff T. Community Coalition Building—Contemporary Practice and Research: Introduction. American Journal of Community Psychology. 2001;29(2):165–72. doi: 10.1023/A:1010314326787 11446274

