# Supplementary material for: Opportunities and challenges in utilizing community assets to extend chronic care management in podoconiosis endemic areas: Evidence from Northwestern Ethiopia
Source: PLoS One. 2024 Oct 22;19(10):e0309770. doi: 10.1371/journal.pone.0309770 (PMC11495552; doi:10.1371/journal.pone.0309770)
Supplement: S1 File — (DOCX) [file pone.0309770.s001.docx]

# Supplementary material

*Opportunities and Challenges in Utilizing Community Assets to Extend Chronic Care Management in Podoconiosis Endemic Areas: Evidence from Northwestern Ethiopia*

Kibur Engdawork^^[[1]](#footnote-1)^*^, Gail Davey^^[[2]](#footnote-2)^,3^, Getnet Tadele^1^, Papreen Nahar^2^, Shahaduz Zaman^2^

College of Social Sciences, Addis Ababa University, Ethiopia. ^2^Centre for Global Health Research, Brighton and Sussex Medical School, UK. ^3^School of Public Health, Addis Ababa University, Ethiopia

* Correspondence:

[Kibur.engawork@aau.edu.et](mailto:Kibur.engawork@aau.edu.et)

## Thematic text extract selection

| **Construct** | **Key findings** | **Supporting quotes** |
| --- | --- | --- |
| Agents  Agents are individuals with ability and potential to make changes in health behavior. | The study identified agents including patients, family members, community leaders, traditional healers, religious leaders with the potential to extend chronic care interventions in the communities.  Family members of affected individuals are sympathetic to patients.  Health professionals have collaborated with religious leaders in prior health interventions  Affected individuals seek help from traditional healers  Traditional healers understand the nature of podoconiosis and provide treatments similar with lymphedema management guidelines  Gender inequality at home pose a challenge to utilize family members in interventions  Health extension workers (HEWs) are highly respected by community members | - “My brothers are sympathetic to my condition. They helped me with agricultural and other activities” (IDI, affected man, age 50) - “My children and my wife take care of me. They wash my feet and suffer with me whenever I get sick. My children would go to the town and buy me pain killers. My wife washes my leg. I become grumpy whenever I have an acute attack, but they tolerate my behaviour” (IDI, affected male, age 56). - “We engage religious and community leaders to extend health information to community members. They are regarded as influential figures. Members respect and implement activities if they are approached via these individuals” (KII, HEWs, Dera district). - “My parents took me to a traditional healer for treatment when I developed podoconiosis. He applied herbal medicine and the pimples on my foot completely disappeared” (IDI, 25 years old affected male). - “To reduce the swelling, I use herbal medicine to wash patients’ feet. I mix the herb with cows’ urine and soak it for a while and apply it on the swollen feet. However, if that does not work, I will move on to the next level of treatment by piercing their feet at various spots with a blade. Then, I will advise them to rinse and soak their feet in the hot spring” (KII, traditional healer, Dera district). - “Whenever I have an acute attack, my husband and children wait till I feel better instead of helping me with my household chores. They would rather go hungry for a day than cook for themselves” (Affected woman, age 45). - “My husband and I are affected by podoconiosis. Whenever he has acute attacks, he sends our children to buy him painkillers. But, whenever I get sick, I sleep it off. My husband and children expect that I would eventually get well without any medication” (IDI, affected woman, age 38) - “Thanks to health professionals we are not using latrines. Back in days we used to defecate wherever we want. Now we even ridicule people who defecate in the fields (FGD, Men Yilmana Densa) - “We are really grateful to HEWs. They work for us. They come and visit us every month. Our children are healthy because of them” (FGD, Women, Dera district) |
| Physical structures and places | Our observations and interviews indicated that households, health posts, schools and healthcare facilities and churches are suitable to conduct chronic care intervention.  Health care facilities provide health services for community members, and they have sufficient places to conduct health education. Intervention implementer utilized the healthcare facilities to conduct health interventions.  Churches have been utilized by prior health interventions. Religious leaders are highly revered by members of the communities.  Prior interventions utilized schools as venues of health education. The schools have sufficient space to conduct health intervention and disseminating messages via schools can be essential to assure social acceptance of preventive messages about podoconiosis  School based intervention may not be sustainable unless health educations are mainstreamed into school systems.  Health interventions may not be successful unless they engage teachers and school administrators. | - We observed two health care facilities serving up to 40,000 residents for surrounding kebeles are found in the study areas. The premises were sufficiently large and contained neonatal intensive care, delivery, TB treatment, examination, emergency, laboratory, drug dispensary and management rooms and waiting areas. Each kebele has one health post accountable to the health center. HEWs operate at health post level. Health extension workers spend some time at health posts providing health education, immunization, and curative services. The health posts are located near to resident areas. They consult patients at health posts. The health centers consist of one physician, five health officers, five midwifes, two laboratory technicians, eight clinical nurses, three pharmacists, and three administrative staff. - “We serve about 1,600 population in this kebele” (KII, HEW, Dera districts). - “We serve about 40,000 residents of the surrounding *kebeles,* and operated with one physician, five health officers, five midwives, two laboratory technicians, eight clinical nurses, three pharmacists, and three administrative staff” (KII, healthcare facility director, Yilmana Densa District) . - “Most of us now go to health centres when we are sick and for delivery. We also go to health posts to get family planning counselling” (FGD, Women, Yilmana Densa District). - “When there was an intervention on podoconiosis, many affected individuals, including those with swollen legs came to the stations to receive treatment” (KII, health professional, Yilmana Densa District). - “The health staff at healthcare facilities soaked our feet in water in a plastic bowl and showed us how to wash our feet” (IDI, a 25-year-old affected male). - “I have seen health professionals in the healthcare facility washing affected individuals’ feet, touching them with their hands. It gave me the lesson that the disease is not transmittable with skin contact” (FGD participant, male group, Dera district). - “Trained staff and HEWs provided health education to the community about podoconiosis at churches. That helped reach a large number of people in the community” (KII, NTD officer, Dera district). - “Most of the children in our area are going to schools these days. The time has changed and every family wants to send their children to school” (FGD, Men, Dera Districts) - We observed that there were two primary schools in the communities. Many children are attending primary education in these schools. - “We did invite affected individuals from households, churches and schools. Accordingly, there was high turnout out of patients at the beginning” (KII Podoconiosis focal staff, Dera district). - “Schools are ideal places to provide health interventions. We distributed medicine for diseases like trachoma and conducted health education intervention at nearby schools. You can easily get students and most of them are willing to take part in health interventions” (KII, HEW, Dera District). - “We sometimes go to schools and conduct health campaigns. But those campaigns usually last for a few days; and lessons can be forgotten easily. We should engage teachers and school clubs to reach the wider student population” (KII, Health professional, Dera District - “The community was very hesitant to get COVID-19 vaccination as teachers warned members and students that the vaccines were not safe. The teachers browsed social media and spread false rumours about the vaccine. That made our campaign very difficult as many people believed the teachers” (KII, HEW, Yilmana Densa District). |
| Religiosity | The majority of the community members are followers of Orthodox Christianity. Religion is highly revered and shapes residents’ beliefs about things. Residents observe fasts and go to church every week to worship. Most people try to explain things including disease in supernatural terms. The community gives high regards to the spiritual structures (associations) and their leaders. | - “I am a farmer. During the farming season I wake up early in the morning go to church to salute and praise the Lord with prayer and set the oxen for farming and go to my farm field to plough. Sometimes I may go directly to the farm and give prayed at the church I may find on my way to the farm” (IDI, affected female, age 64). - “We can’t prevent anything if it is the order form God. Anything can happen anytime by the will of God” (IDI, affected male, age 63) - “People are the creatures of God, and no one is different. Therefor, everyone has been treated as normal human being” (IDI, affected female, age 38). - “The Lord gives and lord takes away. God gave me a beautiful foot at birth, and HE took it away. I have not taken remorse against God. I thank my God because he also gave me the strength to cope up the challenges” (IDI, affected female, age 35 to 38 years old). - “I think the best solution is to teach the community through religious institutions and structurers. ..people have more respect to their religion and to the religious leaders” (KII, HEW, Dera District). - “A well-planned community campaign involving religious leaders might be crucial to achieve community-wide adherence to treatments and prevention practices” (KII, staff of an NGO).   “Everything is given from God. God had power over the disease and stigmatizing against patients could result in having similar or other diseases as punishment. So we don’t discriminate against patients. It is shameful to do (FGD, Women, Yilmana densa district)   - “The community members are more considerate to people affected by podoconiosis. Patients have never received any bad treatments form the community members. They live together in harmony and participate in any social life just like any other healthy individual. There is no discrimination against patients. That is one of the good things I observed in our community. In fact, some people might be reserved to come” (KII, HEW, Yilmana Densa District). - “I have lived my age in this community and have never seen any bad treatment against patients affected by disease of any kind. I have not encountered any problem with regards to my illness from my neighbourhoods in my village. This is just a disease that could have happened to anyone in the community. I think the community members understand this situation very well. I have done nothing to deliberately bring and transmit this disease. Therefore, there is no way that the community could expel or avoid me form social life. I lived peacefully and with good social integration” (IDI, Affected Female, age 64) - “As far as I know, no one stigmatizes anybody. All people are treated in the same way. It is not in the nature of our community. I haven’t encountered any of such people. People are the creatures of God, and no one is different. Therefore, everyone has been treated as normal human being. I have never encountered any. We are surrounded by close relatives in my villages. They are considerate towards me. In fact, when I encountered in conflict with a non-relative, they may use bad words to insults me referring to my illness. Other than that, there is nothing serious happened to me so far” (IDI, Affected female, age 49) |
| Changing norms | Shoe wearing practices. The participants argued that shoe wearing practices is becoming a norm among the younger generation, but still most old people walk barefoot. | - “Young people never go bare feet. Only the people older than me who would like to be bare feet. Particularly walking bare feet is commonly observed among women. I mean in terms of proportion it is the highest among women, but men also do the same specially on farms” (FGD, Men, Dera District). - “In the past, there was no shoes at all. It was on considered as an important item. Only a few people were using footwear. After the health extension program came into a picture in late 1990’s, people started to wear shoes. people started to consider shoes as important item. It is improving but still the practice is inconsistent. Specially people in the rural community do not wear shoes at home or around villages and in farm activities. Most people wear shoes when they travel long distance. In fact, young people are better in this regard, but not conclusive. Still many young people also spend bare feet at home and in the farm fields”(KII, regional health bureau officer, Dera District) - “I think there is a lot of change in shoes wearing practice among the youths and adults. Old people stick to their old tradition and walk bare feet. Improvements in the economic capacity of families has also contributed to the change in shoes wearing practice” (FGD, Women Dera District). - “Today there is improvement with regards to the shoes wearing practices among young people. Except during the rainy season (where almost all people opt to walk bare feet when it is muddy, assuming not comfortable to work) young people wear shoes all day and every day” (KII, HEW, Dera District) |
| Changed practices through community-based interventions. | Experience of communities in community base interventions. The study found that the communities had some experience in community-based intervention that brought normative changes.  From homebirth to health facility delivery. Respondents believe that women in the community are delivering at healthcare facilities following the health education and follow-ups by HEWs.  Seeking treatment from health care facilities. The respondents believed that residents are increasingly visiting healthcare centers to seek treatments.  From open defecation to latrine  Community members reported that defecating in fields and forests had been a common practice as there was a lack of toilets.  Improvement in women status.  Community members reported that women’s right have been improved recently following legal interventions. | - “A number of women used to give birth at home and some died due to heavy blooding during labour. We can say this has been changed now as women give birth at health stations “(FGD, Women Yilmana Densa) - “Unlike the previous times, people now have a community-based health insurance and never hesitate to come to the health center whenever they feel pain. Earlier, people hardly visit the health center except for sever cases. Now they visit the health center for any type of disease and could be referred to higher level medication at woreda or in big towns like Bahirdar”( FGD, Women, Yilmana Densa District). - “Thanks to health professionals we are using latrines. Back in days we used to defecate wherever we want. Now we even ridicule people who defecate in the fields” (FGD, Men Yilmana Densa). - “We have successful end open defecation practice in our community. We worked with community representatives, religious people and health professionals to changes people’s habit of defecating in the field. We constructed latrines in group and those who do defecate on the field were mentioned and were given fine at social gatherings. Through time, everyone is used to use latrine” (KII, NTD officer Dera district). - “Nowadays, women have the right to acquire and use lands and properties. This is because government officials and religious people have been telling community members about women’s right. We have been told about this during idir and equp gatherings. If women get divorced, they will take equal share of the lands and properties. Women have right to hold the right of child custody after divorce. Back in days ex-husbands didn’t support ex-wives. Nowadays every divorced husband financially supports their ex-wives. This is due to law”(FGD, Women Dera District) |
| Established community social support and structures | Most of the affected and unaffected individuals are members of traditional/social institutions such as *idir* and *mahiber.* They believe that their fellow members will extend their help during their difficult times such as a death of a family member.  Idir . the community rely on a self-help voluntary association that serves as economic and social insurance at times of death and other crises. As it stands, idirs only provide help to members during crisis. They don’t serve as platform to help members during sickness or other needs (e.g. clothing) .  Mahiber: Spiritual gatherings under the informal association named mahiber are also common practice in the communities. | - “Everyone in our community is a member in idir. It is established by the community to support and assist people in need specially helping the poor. It is outside the government structure. It involves all the community members” (FGD, Men Dera District) - “Idir is a crucial social institution which is useful to strengthen our social bond. It serves as a social insurance for the poor. In time of death(mourning), idir assists the deceased family equally regardless of their wealth status (FGD, Women Yilmana Densa) - “People are also organized in idir to help each other in time of mourning when someone passes away. The purpose of idir is only to facilitate the funeral ceremony not helping people in other aspects of life. idir provided some money for the family who lost life” (FGD, men, Dera district) - “We commemorate saints on monthly basis under our mahibers (religious associations). This is for our soul and body. By doing so, we perform our spiritual role. In addition, we will have intimate relationship. We talked about our personal problems when we meet” (FGD ,Women Dera District). - “I am a member of the association that honors saint Gabriel. I am the only patient in our association. The association has around 40 members. I usually participate, except when I am hit by acute attacks. I stay at home when the acute attack arises. It is helpful specially in time of harvest and farming or building a house. When I request the support from members, they are willing to assist me in labor” (IDI, a 57 years old affected male) |
| Affected individuals’ understanding of podoconiosis and willingness to take part in health interventions | The study found that patients eagerly participated in prior health interventions. Participation in the intervention enhanced their understanding of podoconiosis. Patients have shared the knowledge they obtained from the intervention to community members through informal relationships. | - “When I was informed by a HEWs about the aid for patients, I was very happy to go to the healthcare facility. We were given soaps and washing basin. We have taken a lesson from health professionals on how to care for ourselves. We were informed by health professionals that we should continue applying the treatment procedures at home. And I have been practicing self-care” (IDI, affected male, age 60). - “Affected individuals tell us about the disease. They went to healthcare facilities and received health education. As they know the disease very well, they tell us how we can protect ourselves” (FGD, women, Dera district). - “I am already affected by podoconiosis. I don’t want my children to suffer from the disease. So, I always tell them to wash their feet and wear shoes” (IDI, affected female, age 39). |
| Lack of understanding about the chronic nature of podoconiosis | Some affected individuals did not know that podoconiosis is a chronic illness. Expectation of instant cure from the treatment, high dependency syndrome and misunderstandings about the intervention accounted for many patients to drop out the treatment. The condition reinforced concerns over beliefs about curability of the disease that leverages the stigma reduction. | - “Frankly speaking, the benefit of taking part in health intervention is not so promising. There is only a slight change in the swelling. I gave up on the treatment activities and I don’t usually practice the elevation exercise” (IDI, affected male, age 43). - “I have given up as soon as the soap and ointments are run out...I cannot afford buying soaps leave alone these types of shoes. I am still waiting for shoe support” (IDI, Affected male age 64) |
| Common health problems from the perspective of community members | The study indicated that community members considered TB, trachoma and malaria as the major health problems. | - “People in our community are being affected by various types of disease. In my opinion, the most serious one is Tuberculosis disease. Because it affects many people even before they are diagnosed, and it is highly transmittable and frightening. - TB is the most dangerous disease affecting people and may have lost their life due to this disease. Trachoma is another one which his widely prevalent causing blindness to many people. Malaria is also serious although it is seasonal. Podoconiosis is prevalent but not serious in terms of magnitude and number of affected population . - “Trachoma is another one which his widely prevalent causing blindness to many people. Malaria is also serious although it is seasonal. Podoconiosis is prevalent but not serious in terms of magnitude and number of affected population” - “Malaria is also serious although it is seasonal. Podoconiosis is prevalent but not serious in terms of magnitude and number of affected population” (FGD, men, Yilmana Densa district). - “Malaria is the most widely prevalent and affecting almost every family in our community. It is transmitted by mosquito and reached to everyone. The treatment is not effective. We have received malaria tablet at the health center, but it continues to live in our blood and relapses time and again. In my view malaria is the most widely prevalent disease” - “I agree with malaria and hemorrhoid disease. These two diseases have affected everybody. I was examined last year in the health center, and they found malaria in my blood and gave me treatment” (FGD, women, Dera district)   . |
| Lack of chronic care services for patients | The study revealed that current efforts to provide chronic care services for affected individuals are non-existent. Healthcare facilities only provide services when patients have episode of acute attacks. | - “We usually administer medicine for acute diseases. Even when podoconiosis patients visit us when they have acute attacks, we give them antibiotics and advise them to keep their personal hygiene. We don’t follow their progress and we only try to treat their acute attacks if they come to our healthcare facility” (KII, health officers, Dera district). |
| Physical, psychological and economic impact of podoconiosis | Podoconiosis affects the mobility of patients. Patients also face frequent acute attacks characterized by chills and fever. This affected the productivity of patients and their self-esteem. | - “I was frequently suffering from an acute attack. When acute attack arises, I feel cold inside and pain around the groin. I also sleep the whole day or a couple of days and weeks until the acute attack subsides. That is the worst part of this illness” (Affected Male, age 64). - “It has been four years since I started having frequent acute attack. Every time I face acute attack, I have waist and muscle ache; whole body becomes weak. I develop chills and I suffer like an old person. The acute attack gets worse during the cold season” (Affected male, age 40). - “The main problem I am often encountering with this disease is the acute attack. It interferes with my daily activities. I can’t do my domestic work. I can’t fulfill my responsibility at home. I can’t travel long distance (such as going to the market or visiting relatives in other locations)” (Affected Female, age 65) - “Compared to healthy people, we are useless. My leg doesn’t take me forwards. It holds me back. When we travel as a group to markets or other places, I stay behind. When people reach to their destination, I could only reach the middle” (Affected male, age 39) - “I am a farmer… The disease is painful and sometimes it keeps me stay at home. During the farming season it leaves me behind from preparing the farmland for the next harvest. Missing out that period affects my economic life.” (IDI, male,50 years old). |
| Political context | Ethiopian government has shown commitment to fight podoconiosis. Podoconiosis has been included national program and masterplans. The inclusion of podoconiosis in the national strategic plan is important as NGOs and funders could be encouraged to fund and work on interventions that area part of the national strategic plans. The regional government demonstrated its commitment in setting up the NTD structures at zonal and woreda levels. NGOs working on podoconiosis prevention and control reported that government offices collaborated with them during implementation of interventions. However the government doesn’t back this with allocating budget.  . | - The Ethiopian Ministry of Health plays a leading role in mental health leadership and governances. The ministry is responsible for ensuring the sustainable supply of medicine, securing financial resources and strengthening the research, information, monitoring and evaluation systems for mental health. In addition, the ministry develops and revises national plans and identity target of interventions (FMoH, 2016). In line with the country’s political administrative structure, the mental service delivery system is governed by the Federal Ministry of Health at national level and regional health bureaus at regional level. The mental health program is placed under the Disease Prevention and Control Directorate in the MoH with focal persons for mental health in the district health offices (FMoH, 2016). Podoconiosis finally became a national priority in 2013 and was included in the National Master Plan for Neglected Tropical Diseases (NTDs). The Master Plan was developed in order to provide strategic direction for the implementation of the prevention, control, and elimination of podoconiosis and other NTDs (Ministry of Health, 2016). - The Ministry of Health developed integrated Lymphatic Filariasis and Podoconiosis Morbidity Management and Disability Prevention Guidelines (Ministry of Health 2016). These guidelines provide evidence based practical guidance on patient management and disability prevention. At grassroot level (Ministry of Health, nd.). - The Government of Ethiopia has also developed a National Hygiene and Environment Health (HE) and Neglected Tropical Diseases (NTD) Message Guide to support NTD services. The guide is meant to communicate core messages regarding podoconiosis treatment and prevention methods with the intention of diffusing consistent messages across various communication channels (Ministry of Health, 2018). - Regional offices always collaborate with us. This has enabled sharing, knowledge, resources and experience, and minimized resource duplications” (KII, staff of intervention implementing organization). - The NTD section has been established in the health sector structure at regional, zonal and district level to account for these neglected health problem.” (KII, NTD officer, Yilmana Densa District). - “The Ministry has not allocated any budget for podoconiosis in the years 2022 and 2023” (KII, Podoconiosis focal person at Ministry of Health). |
| Lack of sustained health services of patients. | The study found that a prior health intervention known as “next steps for podoconiosis patients in Amhara region” provided some health resources for patients and conducted health education intervention in the communities. The intervention was conducted using healthcare facilities in a bid to mainstream the activities into local healthcare services.  It was assumed that mainstreaming podoconiosis in the NTD structures and health centers reinforce sustainability of self-care treatment and ultimately improve the physical, social, and economic wellbeing of patients including stigma reduction. However, the activities could not be sustained at the healthcare facilities. | - The aim of the project was to ensure and strengthen integration of the podo operation in the target health centers” (KII, IOCC staff) - Integration was taken as a means for sustainability as it provides opportunity for creating access and open doors services for patients, which was also believed to eventually contribute to stigma reduction (KII, District NTD officer) - “We once provided health resources and treatment for podoconiosis patients for three months. However, we could not continue the services as our healthcare service is not receiving support from the health bureau” (KII, health professional, Yilmana Densa District). - “Although I went to a healthcare facility this year and asked the health staff to register me for support, they informed me that it was too late, and no service was provided at that time” (IDI, an affected female, age 38). |

## Appendices

In-depth Interview (IDI) guide with Individuals affected by Podoconiosis

1. Would you please tell us about the prevalent disease in your community?
2. Can you please describe how you developed podoconiosis (What do you say the causes are)?
3. Have you sought any treatment for your condition (What healthcare institutions have you visited? Where did you go first? What progress have you made?)?
4. How do you describe being a patient of podoconiosis?
5. What were the major challenges you have faced after you developed the condition?
6. Tell me about the individuals with whom you have a regular interaction with, people who are close to you, people to whom you feel close, people with whom you discuss important matters, (names, role relations (family, friends etc.), how often do they interact?
7. What was the reaction of these individuals after you developed the condition?
8. How do you evaluate your participation in religious and traditional institutions in your community?
9. What are your future plans (education, work, marriage, etc.)? Do you have any concerns that you may not achieve your plans due to the disease? How or Why?

Please elaborate on the impact of podoconiosis on your daily life – Social relations (family, friends, partners), , support, psychological (at work, - paid/unpaid).

1. How do you explain if because of your conditions you were treated differently at home, at neighborhood, at school, at religious/social ceremonies or festivities?? what negative experiences you have? (please list all experiences at the different levels) Why is this the case?
2. What have you done to overcome the stigma you have faced due to your condition?
3. Who do you think are most vulnerable to podoconiosis?
4. Where did you get information about podoconiosis?
5. What do you think most people think about podoconiosis in your locality (what they think about its causes, prevention and treatment? What words do they use to describe the condition and patients)?
6. Tell me about your involvement in interventions that aim to control podoconiosis (When did you start getting involved in the program? How did you come to be involved in the project? What are the activities you have been participating in? What is your opinion about the activities?).
7. What benefits do you get as a result of participating in the program?
8. What changes do you think interventions have brought in improving the lives of patients?
9. What do you think should be done to improve the lives of affected individuals and to control podoconiosis?

Focus Group Discussion (FDG) Topic Guide with Community Representatives

1. What are the prevalent health problems in the community?
2. What modern and traditional healthcare institutions are there to deal with the health problems?
3. What is the common thinking about podoconiosis in this area.? (Perceptions about the causes and prevention of podoconiosis, what term is used to refer to the condition?)
4. What sorts of norms and practices are there in this community to fight health and social problems ?
5. Have these norms and practices shifted over time? If so, how and why?
6. Has there been any attempt by the community to change these norms (what changes have been observed with regard to people’s capacity to adopt healthy behaviour)?
7. Lets’ discuss now on programs that are implemented to control and prevent podoconiosis other social problems in this area (who are they? How do they operate? Does their opinion carry weight in the community? why?)
8. What sorts of norms and practices are there in this community around wearing shoes? (Do people wear shoes regularly, if not why? Are there any differences between men and women?)
9. Have these norms and practices shifted over time? If so, how and why?
10. What are the most important social and economic institutions in this community? (What types of benefits do these institutions offer to their members? When and how do they offer these benefits? How to you see the involvement of these institutions in health promotion interventions? )
11. What are the criteria for membership?
12. And, what are the most important values, customs, traditions dearly/greatly upheld by these religious and traditional groups?
13. What are the challenges and opportunities facing community organizations such as schools, traditional and religious groups?
14. How is the participation of patients of podoconiosis and other individuals in these institutions (Are there any ideas/customs/attitudes that discourage patients of podoconiosis from participation in the institutions?
15. Are there specific people/groups known for limiting the participation of patients of podoconiosis in these institutions (who are these people? Why does their opinion matter? Why are they influential?)
16. What do you think should be done to control podoconiosis and other health conditions?

Key informant interview guide

1. Would you offer me some historical background on how the district assumed its present political status

2. Would you tell me the current organization and structure of the district level (legislative, judiciary and executive organs)?

3. What are the unique characteristics particularly attributable to the district within regional or national context (for e.g. Known for its mineral resource, natural resource etc.)?

4. What are the major economic activities in the district?

5. What percentages of the population are engaged in these major activities?

6. What public services (electricity, telephone, water, road etc.) are available in the community?

7. How accessible are these services to the community (how satisfied or dissatisfied are community members with regard to the provision of services)?

8. What are the customs and practices related to shoes wearing (do people wear shoes on a constant basis, if not, why? Are there any differences between men and women in shoe wearing practice?

9. Have these customs and practices shifted over time? If so, how and why?

10. Please tell in terms of priority, that you consider are the unmet needs of the community that deserve special attention?

11. Would you tell me the availability and distribution of public or private social services in the district (schools, vocational college, heath institutions, etc.)?

12. Would you tell me about the major health problems in the district?

13. What has been done to deal with the health problems (what are the major achievements? What challenges or barriers have been observed in dealing with health problems?

14. What has been done to control and prevent podoconiosis in the community (What changes have been brought by this effort? Is this enough? Were there other things that should have been done? By whom? Why weren’t they done? What needs to improve for these to be done?)

15. What do you think about NGOs that implement health-related intervention in the district? (what are the major changes brought about by the projects?)

16. What do you think should be done to make health promotion efforts more effective and sustainable?

1. [↑](#footnote-ref-1)
2. [↑](#footnote-ref-2)
